# The Pain Situation of Older Home-Care Recipients and Influencing Factors

**DOI:** 10.1093/geroni/igaa057.662

**Published:** 2020-12-16

**Authors:** Dagmar Dräger, Reinhold Kreutz, Adelheid Kuhlmey, Andrea Budnick, Dagmar Draeger

**Affiliations:** Charité - Universitätsmedizin Berlin, Berlin, Berlin, Germany

## Abstract

Chronic pain is a common symptom among older people. The international prevalence rate reaches 50% for older home-care recipients (aged ≥60). The most common causes of pain among older people are degenerative arthropathy and musculoskeletal diseases. Care recipients (81% aged ≥65) constitute a specific sub-group among pain patients, due to the restrictions they experience. In Germany, the prevalence rate in this group is 70%. Currently, no comprehensive information on the pain situation of older home-care recipients exists in Germany. The findings presented are based on a cross-sectional study of older (aged ≥65) home-care recipients (SGB XI) in Berlin, with chronic pain (n=225), capable of self-report (MMST≥18). Structured interviews comprised the primary data source. The pain situation was determined using the German Brief Pain Inventory (BPI-NH). Multiple regression analysis was applied to test how the most severe pain (dependent variable) was influenced by socio-demographic and medical parameters, mental and physical restrictions and pain medication. Analyses of the pain situation show a value of M=4.81 (SD±1.88) on the BPI intensity index, and a BPI pain interference index of M=5.47 (SD±2.15). The most intense pain averaged 6.96 (SD±2.15). On average, respondents reported 16.20 (SD±13.25) pain locations (range: 0-65). The number of pain locations, alongside other factors, had a significant influence, R²=0.038 (corrected R²=0.034), F (1.219) = 8.760, p<0.01), on pain intensity. The findings show severe pain intensity among older home-care recipients not reported in previous findings (e.g. in long-term in-patient care). Action in medical care, nursing care and educational aspects is urgently needed.

